# Sleep Physiology Alterations Precede Plethoric Phenotypic Changes in R6/1 Huntington’s Disease Mice

**DOI:** 10.1371/journal.pone.0126972

**Published:** 2015-05-12

**Authors:** Fanny Lebreton, Sebastien Cayzac, Susanna Pietropaolo, Yannick Jeantet, Yoon H. Cho

**Affiliations:** 1 Institut de Neurosciences Cognitives et Intégratives d'Aquitaine, CNRS UMR 5287, Bat B2—Avenue des Facultés, 33405 Talence Cedex, France; 2 Université de Bordeaux, Bat B2—Avenue des Facultés, 33405 Talence Cedex, France; University of Oxford, UNITED KINGDOM

## Abstract

In hereditary neurodegenerative Huntington’s disease (HD), there exists a growing consideration that sleep and circadian dysregulations may be important symptoms. It is not known, however, whether sleep abnormalities contribute to other behavioral deficits in HD patients and mouse models. To determine the precise chronology for sleep physiology alterations and other sensory, motor, psychiatric and cognitive symptoms of HD, the same R6/1 HD transgenics and their wild-type littermates were recorded monthly for sleep electroencephalogram (EEG) together with a wide range of behavioral tests according to a longitudinal plan. We found an early and progressive deterioration of both sleep architecture and EEG brain rhythms in R6/1 mice, which are correlated timely with their spatial working memory impairments. Sleep fragmentation and memory impairments were accompanied by the loss of delta (1-4Hz) power in the transgenic mice, the magnitude of which increased with age and disease progression. These precocious sleep and cognitive impairments were followed by deficits in social behavior, sensory and motor abilities. Our data confirm the existence and importance of sleep physiology alterations in the widely used R6/1 mouse line and highlight their precedence over other plethoric phenotypic changes. The brainwave abnormalities, may represent a novel biomarker and point to innovative therapeutic interventions against HD.

## Introduction

Huntington’s disease (HD) is a hereditary neurodegenerative disease with dominant autosomal transmission. The disease is caused by a prolonged CAG repetition in the gene encoding for huntingtin protein [1]. While the mutated Huntingtin protein is widely expressed, neuropathological signs are detectable early in caudate-putamen [2]. The abnormalities spread into the entire brain at late stage of the disease. Among phenotypic changes observed in HD patients, cognitive (visuo-spatial learning, cognitive flexibility) and psychiatric (impulsivity, depression, anxiety, social isolation) symptoms often precede choreic and motor abnormalities. Circadian rhythm dysregulation and sleep disturbances are also important early symptoms in both patients [3,4,5] and mouse models [6,7,8]. Because sleep has long been associated with off line information processing and memory consolidation [9,10,11] in addition to many behavioral and physiological functions, the sleep-wake cycle dysregulation is expected to exert an important role in cognitive abilities in humans and mice.

The previous sleep studies in transgenic mice employed mainly actimetry analyses based on wheel running and cage activity [6,7,12], and only recently a few electroencephalogram (EEG) recordings have been conducted during sleep-wake cycles in the R6/2 and R6/1 mouse models of HD [13,14,15]. While these EEG studies confirmed sleep fragmentation [13,14] and early changes in the EEG [13,14,15] in these mice, they did not concomitantly evaluate cognitive and behavioral disturbances to define the precise chronology for sleep physiology alterations and behavioral symptoms of HD. For example, the previous R6/2 mice that express about 250 CAG repeats, instead of 160 repeats in the original R6/2 line [16], have not been subject to detailed evaluation of their behavioral phenotypes.

This study thus aimed (1) to verify if changes in sleep architecture and/or brain rhythms during sleep-wake cycles also exist in the R6/1 mouse line, i.e. a mouse model of HD with late disease onset and milder phenotypic progression, and (2) to relate these sleep characteristics to sensory, motor, cognitive and psychiatric perturbations in the same animals. We therefore performed repeated electrophysiological recordings during sleep-wake cycles together with behavioral assessments over the period of onset and progression of the pathology according to a longitudinal plan. Even though only a fragment of the whole huntingtin protein is expressed, we chose to study the R6/1 transgenic line with juvenile onset rather than other Knock-In models with greater construct validity, since the latter are characterized by more subtle phenotypes, appearing only at very advanced ages. Indeed a late disease onset and slow disease progression would not have fit with the time requirements for a longitudinal study with electrophysiological recordings.

## Materials and Methods

### Ethics Statement

Experimental procedures reported here were approved by the local Institutional Animal Care and Use Committee (Comité d’Ethique pour l’Expérimentation Animale Bordeaux), and followed European Communities Council Directive of 24 November 1986 (86/609/ EEC).

### Subjects

Subjects were male R6/1 (B6.Cg-Tg (HDexon1) 61Gpb/J, Stock number: 006471, Jackson Laboratory, Main Harbor, NY, USA) and age-matched wild-type (WT) littermates [16] which were bred and genotyped as previously described [17,18]. While the CAG repeat size was not determined for the mice used in this experiment, the number in the breeders was 123.64 ± 0.89. Mice weaned at 21 days of age were group-housed with their same-sex littermates (3–5/cage) until the beginning of experiments. NMRI female mice (Janvier, Le Genest-Saint-Isle, France) of 3–4 months of age were used as stimulus animals in the social tests. Each stimulus animal was used 3–4 times in the same experiment; its use and order of presentation was always balanced across genotypes. All animals were housed in unisexual groups (3–5 per cage) in polycarbonate standard cages (33x15x14 cm in size; Tecniplast, Limonest, France), provided with sawdust bedding (SAFE, Augy, France), and a stainless steel wire lid. Food chow (SAFE, Augy, France) and water were provided *ad libitum*. The animals were maintained in separate male and female identical colony rooms under temperature (22°C) and humidity-controlled (55%) conditions with a 12:12 hr light—dark cycle (lights on at 7 a.m.).

### General behavioral procedures

Three different cohorts of mice with equivalent numbers of WT and R6/1 (TG) mice were monthly tested according to the longitudinal plan described below (Table 1). They were selected randomly from 16 litters and were naïve to all tests. A first cohort of 14 (5 WT and 9 R6/1) mice were submitted only to behavioral tests, they were 9–10 week-old at the beginning of the behavioral studies. A second cohort of 10 (4 WT and 6 R6/1, 8–9 week-old at surgery) mice underwent both behavioral and electrophysiological recordings. An additional third cohort of 12 mice (5 WT and 7 R6/1 of 8–9 week-old at surgery) was submitted to only electrophysiological recordings. All mice of the three cohorts were housed individually during the entire period of experiments (8–32 weeks) and their health was monitored daily. All behavioral tests, except for sleep recording, were performed on the light cycle.

**Table 1:** Three cohorts of R6/1 and littermate mice used.

Cohort #	Number of mice	Number of dead mice (age of death, weeks)	At 8–9 weeks of age	At 10–11 weeks and monthly
1	5 WT, 9 R6/1	3 R6/1 (18, 18, 20)	No surgery	Only behavior
2	4 WT, 6 R6/1	2 R6/1 (22, 23)	Implantation surgery	Both behavior and sleep recording
3	5 WT, 7 R6/1		Implantation surgery	Only sleep recording

For the cohorts # 1 and 2, body weight, clasping as well as inverted grip test were measured on day 1, with at least a 30 min interval between the tests. On day 2 the rotarod test was performed (3 sessions 1 hour apart). On day 6, the animals underwent a direct social interaction with an adult female, followed on day 8 by an olfactory discrimination test. From day 9 to day 19, electrophysiological recordings were conducted on the cohort #2 (one mouse per day). On day 20 all mice from both cohorts were food deprived and kept to the 85–90% of their *ad libitum* body weight; they were also habituated to the T-maze for three days. Finally, from day 24 to day 27, the reinforced T-maze alternation task was conducted during 4 daily sessions. Following the last T-maze session, mice were again provided with food and water *ad libitum* for 4–5 days (i.e., the time required to recover normal weight) before starting again the tests. This sequence of tests was repeated 4 times, beginning at 10–11 weeks until 26 weeks of age. The third cohort of mice was submitted to only electrophysiological recordings performed monthly from 9 to 26 weeks of age. At 30 weeks of age, all mice were euthanized (mixture of ketamine (100mg/kg) and xylazine (10 mg/kg)) and gently perfused transcardially with 100 ml paraformaldehyde 4% for histological study.

### Body weight, clasping, inverted grid test and rotarod

Body weight of all mice was assessed monthly from 4 weeks of age. For the tail clasping test, mice were suspended by the tail for 10 s; if the mouse acquired a locked body position the result was scored as positive [16]. For grip strength, each mouse was placed on a standard runged cage lid, which was then shaken gently inducing the mice to grip the rungs, prior to rotating 180° through the horizontal plane. The latency to fall, or a maximum of 60 s, was recorded. Three trials were administered with 30 min inter-trial interval. Motor coordination and balance of mice were assessed in the standard accelerating rotarod apparatus (LE8200, Panlab S.L. Harvard Apparatus, Barcelona, Spain), with the rotating speed increasing from 4 to 40 rpm during the 5-min trial. Mice were given 3 testing trials with at least 1 hour interval. The latency to fall from the beam, with a maximum of 5 min, was recorded.

### Direct social interaction with an adult female

Subjects encountered an unfamiliar stimulus mouse (a 12-week old NMRI female) in their cage during a 3 min-session. Video as well as ultrasonic vocalizations (USVs) were recorded and analyzed as previously described [18,19]. An observer unaware of the genotype of the animals scored the time spent performing social affiliative (sniffing the head, snout and anogenital part of the body of the partner) and nonsocial (rearing, selfgrooming and digging) behaviors.

### Olfactory habituation/dishabituation test

The olfactory habituation/dishabituation test [20] consisted of sequential presentations of different odors (water and 2 non-social odors, 3 trials for each odor) to the animal. Different odor-pairs (lavender vs orange, raspberry vs orange flower, lemon vs cinnamon, vanilla vs rose) were used for four different ages tested and the order of presentation was counterbalanced across genotypes. Experimenter naïve to genotype scored sniffing of cotton applicator, which was spread with odor, using stopwatch. Habituation is defined by a progressive decrease in olfactory investigation (sniffing and exploration) towards a repeated presentation of the same odor stimulus. Dishabituation is defined by a reinstatement of sniffing when a novel odor is presented.

### Reinforced spatial alternation task in a T-maze

The apparatus used for this test was a modified T-maze constructed of wood, and similar to the one described by Ainge et al [21]. Briefly, the maze contained one central (50x10 cm), one perpendicular corridor on which formed two goal arms, and two connecting arms (50x10 cm) which linked the distal extremities of the goal arms to the beginning of the central corridor. The maze was equipped with five removable doors. Three of them separated a starting compartment at the beginning of the central arm, by closing the access to the connecting arms and to the central corridor. The other two doors were placed at the entrance of each goal arm. Food deprived mice were submitted to 4 daily training sessions (30 trials). Mice were reinforced (10μl of sweeten milk) by alternating between the two arms, and punished for their errors by being confined in the connecting arm for 10s. Data were expressed as % correct trials for daily sessions.

### Electrodes and Surgery

Mice of cohorts # 2 and 3 were chronically implanted with an 8-electrode multisite array under stereotaxic surgery as described previously [15,17]. Two electrodes held in a stainless steel tube (0.4 mm dia) were positioned approximately 1.5 mm (motor cortex) and 3.0 mm (striatum) below the cranium at 0.7 mm anterior to Bregma and 1.4 mm to the right of the midline suture. Three electrodes inserted in a second stainless steel tube were positioned at 1.9 mm posterior to bregma and 1.4 mm right of midline, and approximately 1.2 mm (sensory cortex), 1.7 mm (CA1 of the hippocampus), and 3.5 mm (thalamus) below the cranium. These depth electrodes for local field potential (LFP) recordings were made of a single strand of 25 μm (dia) NiCr wire [15,17]. Two additional surface electrodes (50 μm-diameter NiCr wire) for EEGs were positioned in the left frontal lobe (2 mm anterior to Bregma and 1 mm left of sagittal suture) and in the central area of the cerebellum (1 mm posterior to Lambda). Finally, a 50 μm-diameter NiCr electrode was positioned in neck muscle for electromyogram (EMG) recording. Stainless steel tubes containing electrodes were used as the animal ground and reference electrode [15].

Implantation surgery was conducted under Ketamine (50mg/kg) and Xylazine (100mg/kg) anesthesia. They were given analgesic and anti-inflammatory agent (carprophene, 10 mg/kg) during and following surgery, and allowed to recover from surgery for at least a week. Mice were then handled daily and habituated to the recording room for 48h and the chamber for 24hr prior to recording. For the cohort #2, recording started at 10 a.m. and lasted for 23h for the two first recordings (at 11 and 16 weeks) and lasted for 4h for the following recording sessions. The third cohort of mice was recorded only for 4h monthly from 9 to 26 weeks of age.

Electrophysiological recordings were performed using Sciworks software (Datawave Technologies, Loveland, CO). Signals were amplified (x 2k), bandpass-filtered (0.1–475 Hz) (Neuralynx, Bozeman, Montana, USA) and sampled at 2 KHz (Sciworks, DataWave Technologies, Loveland, CO). The animal's position also was tracked at 50 Hz with a video camera system (Camera tracker, DataWave Technologies, CO) which followed an infrared lamp placed on the headstage. Following the experiments, Thionine staining of brain sections was used to examine depth electrode localization.

### Electrophysiological data Analysis

Recorded LFPs and EEGs as well as electromyogram (EMG) for all mice and ages were first visualized for inspection, and each 10 s epoch was scored as wakefulness, slow wave sleep (SWS) or Rapid Eye Movement (REM) sleep according to well established criteria [15,22] for the construction of hypnograms. An experimenter naive to age and experimental group performed this operation. The specific LFP recording during each vigilance state for each animal and age was then processed for power spectral analysis [15,17] using Fast Fourier Transforms performed on 4 s Gaussian windows with 50% overlap. Because the intensity of the spectra could be different among electrode probes and animals, for the purpose of comparison among genotypes and ages, intensities for different frequencies were normalized by the sum of the overall spectra (between 0.5–120 Hz) before being submitted to averaging by genotype or age. A part of electrophysiological recording data has been reported in our recent work [15]. Because our previous report described spectral densities of >20 Hz in both genotypes, the present report focused on delta (1–4 Hz) and theta (4–8 Hz) frequencies.

### Statistics

Because no significant difference (effect of “electrode implantation” factor) was observed between the cohort #1 and the cohort #2 for all behavioral measures, their data were combined for analyses. Behavioral and sleep spectral data were analysed using multivariate ANOVA and further post-hoc Student’s *t* test. Mean episode duration, number and total percent time spent in waking (W), slow wave sleep (SWS) and REM sleep (REM) during both light and dark cycles were analysed using non-parametric Mann-Whitney test. The significance was set at *p*<.05 for all tests and the data were presented as mean +/- SEM.

## Results

### Sleep hypnograms

**Fig 1:**
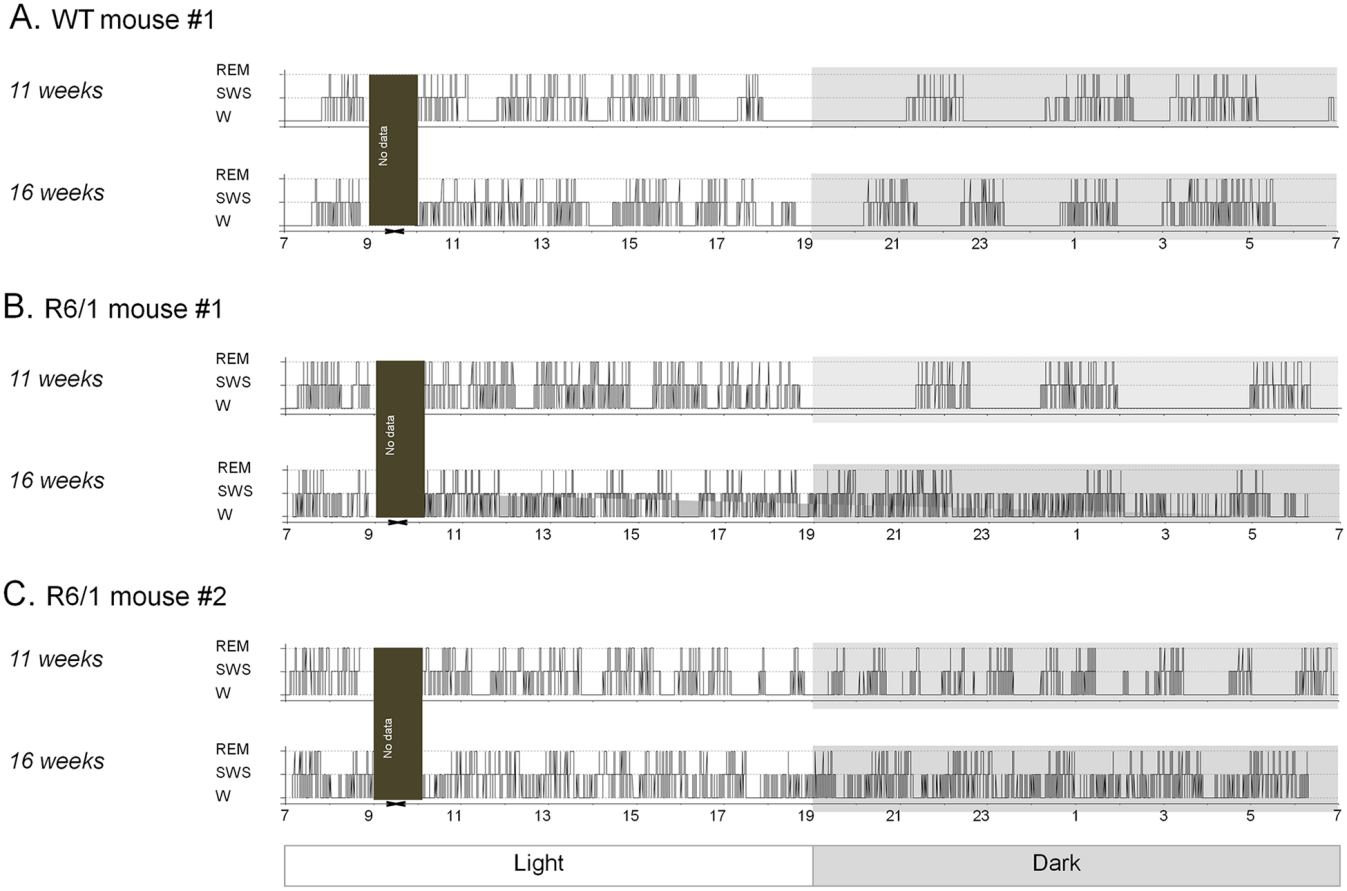
Example of sleep hypnograms of one WT (A) and two R6/1 mice (B-C) at 11 and 16 weeks ages. W: waking, SWS: slow wave sleep, REM: REM sleep.

Fig 1 shows sleep hypnograms of a WT mouse and two R6/1 mice. WT hypnograms at both 11 and 16 weeks were characterized by long time spent awake during the dark cycle (Fig 1A). During the light cycle, such prolonged waking was replaced by frequent sleep-waking transitions and an increased sleep time. R6/1 mouse #1 at 11 weeks exhibited sleep-wake architecture which is indistinguishable from that recorded from the previous WT mouse of both ages (Fig 1B). However, the same transgenic mouse at 16 weeks did not anymore display sustained waking at dark cycle as it did at 11 weeks. Sleep hypnogram of another R6/1 mouse (#2, Fig 1C) displayed disintegrated sleep-wake pattern already at 11 weeks, with increased sleep episodes and shorten waking durations at dark cycle. Reason for this difference between the two transgenic mice is presently unknown.

**Fig 2:**
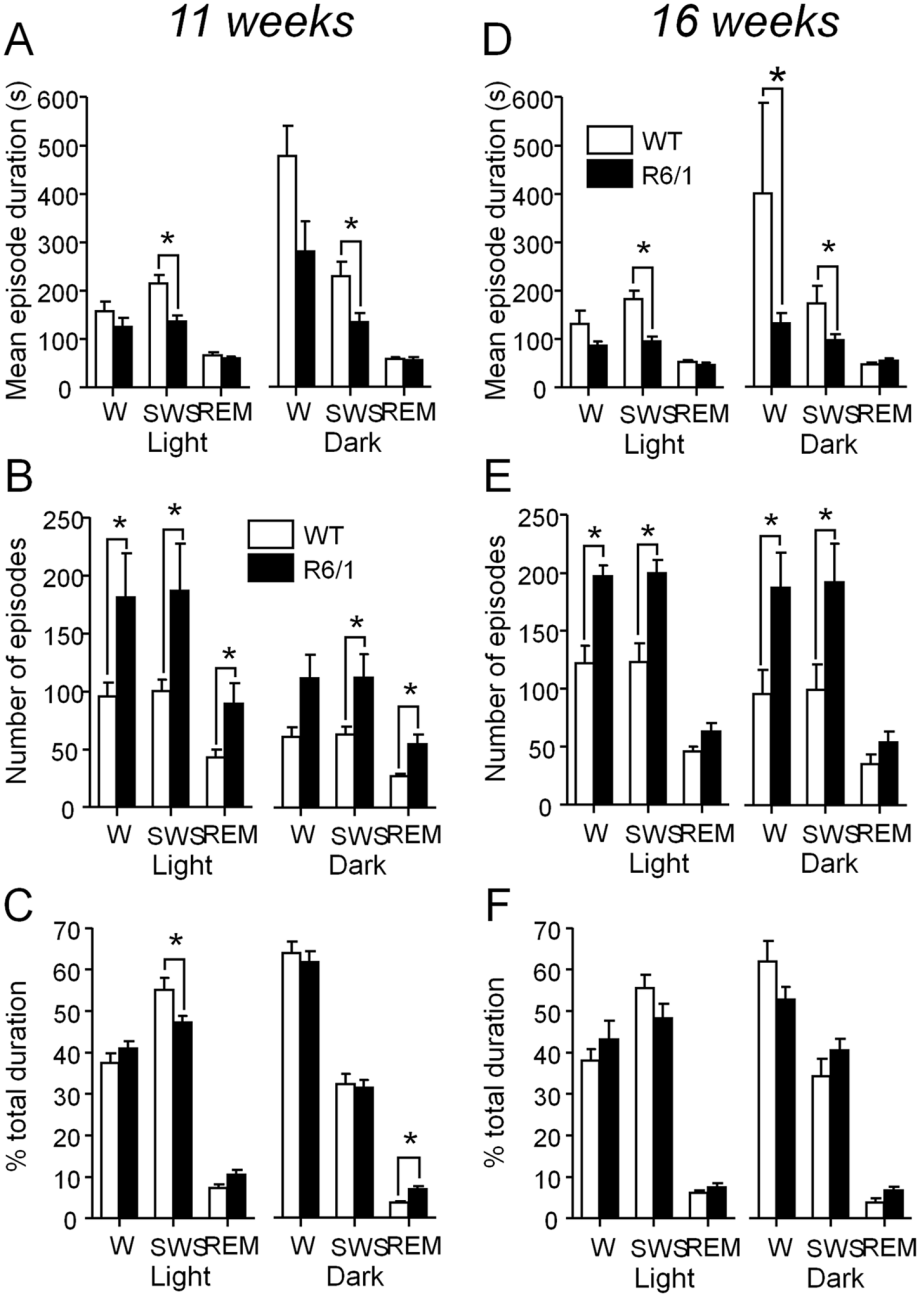
Sleep parameters recorded, in both genotypes, at 11 (4 wt and 6 R6/1 mice) and 16 weeks (4 wt and 5 R6/1 mice) of age. Mean episode duration (A, D), number (B, E) and total percent time spent (C, F) in waking (W), slow wave sleep (SWS) and REM sleep (REM) in both light and dark cycles. * R6/1 mice significantly different from age-matched WT littermates at *p*<.05 by Mann-Whitney test.

Statistical analysis of quantitative sleep parameters confirmed previous qualitative observations of hypnograms and results were summarized in Fig 2. At 11 weeks R6/1 mice displayed a shorter duration of SWS episodes during both light and dark cycles (*p*<.05 for both comparisons, Fig 2A), and a greater episode number for each of vigilance states during both light and dark periods (*p*<.05 for all comparisons, Fig 2B), except for waking episode number at dark cycle (p>.05). However, increased episodes of sleep and waking, and thus transitions between vigilance states in R6/1 mice did not yield a massive change of total sleep or waking time (p>.05, Fig 2C) except for a significant decrease of SWS duration during light cycle (*p*<.05) and a significant increase of REM sleep duration during dark cycle (*p*<.05).

The increased sleep-wake transitions and thus sleep fragmentation in R6/1 mice worsened at 16 weeks (Fig 2D–2F). Statistical analysis of the same data involving light and dark cycles as main factor showed that mice from both genotypes, at 11 weeks, spent more time awake and exhibited an increased waking duration at dark as compared to light cycle (p<.05 for all comparisons). In addition and despite of increased sleep-wake transitions, transgenic mice spent more time in proportion sleeping in both SWS (*p*<.01) and REM sleep (*p*<.05) during light than dark cycle as did WT littermates (*p*<.05 for both sleep periods), revealing their somewhat normal sleep architecture. At 16 weeks of age, R6/1 mice spent a similar amount of time awake and sleeping at both dark and light cycles (p>.05 for three comparisons).

### Cortical spectral changes during sleep-wake cycles

**Fig 3:**
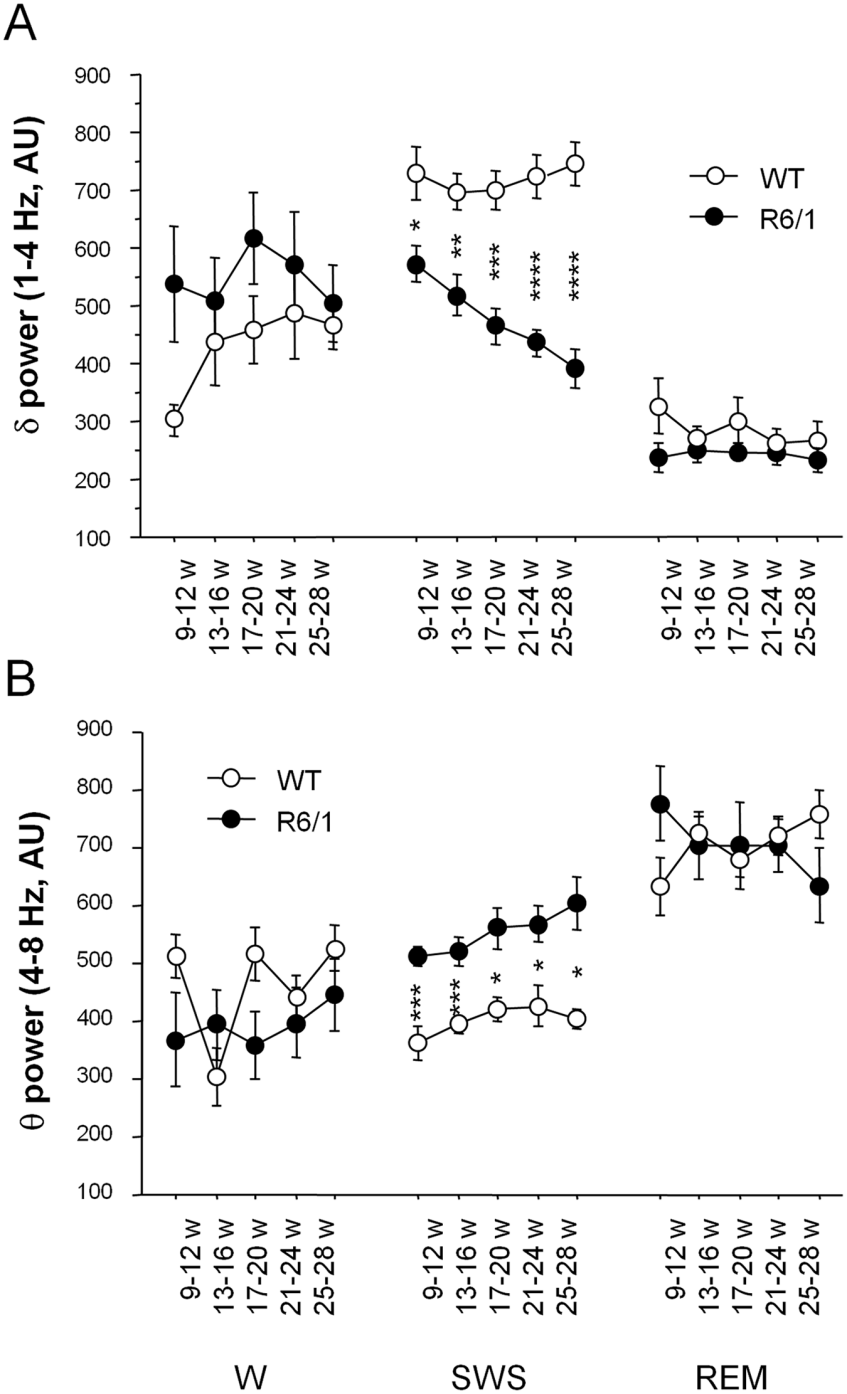
Spectral analysis of recordings during waking (W), slow wave sleep (SWS) and REM sleep (REM) in both genotypes across 5 ages. Averaged summed delta (1-4Hz) (A), theta power (4–8 Hz) (B) at different ages in both genotypes. * R6/1 mice significantly different from age-matched WT littermates at *p*<.05, ** at *p*<.01, *** at *p*<.001, **** at *p*<.0001, AU: Arbitrary unit.

**Fig 4:**
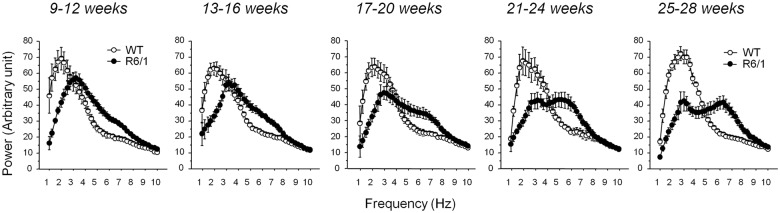
Spectral power for 1-10Hz averaged by genotype during SWS (slow wave sleep) at different ages in both genotypes. AU: Arbitrary unit.

The averaged power spectrum during each vigilance state was calculated and compared between two genotypes at different ages (Figs 3 and 4). Because different LFPs exhibited similar spectra due to volume conduction (Gerbrandt 1978) in the small mouse brain, only data from somatosensory cortex were presented because these were representative of the spectral variation in both genotypes, and hippocampal theta synchrony was detectable at this recording site.

Two-way (genotype and age) ANOVA of spectral variations indicated that in R6/1 mice as compared to WT littermates, delta (summed 1-4Hz) power in general was significantly decreased at SWS (*F*_1,65_ = 82.24, *p*<.0001, Figs 3A and 4), and REM sleep (*F*_1,60_ = 5.05, *p*<.05), while increased at waking (*F*_1,70_ = 4.18, *p*<.05, Fig 3A). Furthermore, the magnitude of delta power reduction at SWS further augmented as R6/1 mice age (genotype x age interaction, *F*_4,65_ = 2.51, *p*<.05).

Contrary to delta power, R6/1 mice exhibited an increased power for theta synchrony (4-8Hz) during SWS (*F*_1,65_ = 56.32, *p*<.0001), and this regardless of age (genotype x age interaction, *F*_4,65_<1, n.s., Figs 3B and 4). Such genotype effect was not found for the other vigilance states (p>.05). Fig 4 illustrates power changes of delta and theta frequency ranges during SWS across five age points, and spectral changes of higher frequencies, specifically in the beta/low gamma (20–35 Hz) range, have been described in our previous report [15].

### Body weight, survival, clasping, inverted grid test and rotarod

**Fig 5:**
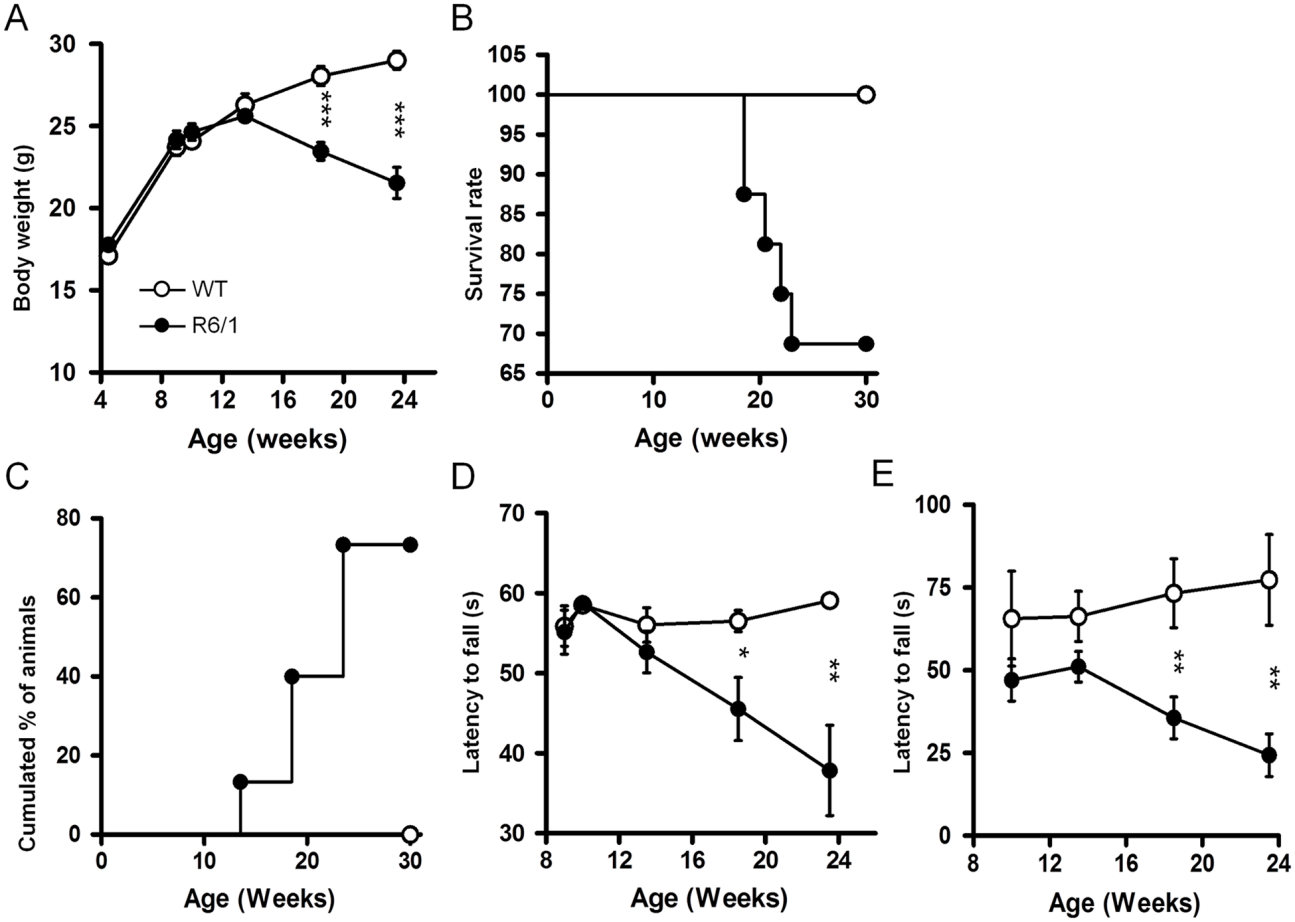
The evolution of body weight (A), survival rate (B), hindlimb clasping (C), inverted grip test (D) and Rotarod test performance (E) in R6/1 mice. * R6/1 mice significantly different from age-matched WT littermates at *p*<.05, ** at *p*<.01, *** at *p*<.001.

As compared to WT mice which continued to gain weight, R6/1 mice began to lose weight at 18 weeks (genotype x age interaction: *F*_5,125_ = 17.08, *p*<.0001, Fig 5A). Transgenic mice weighted significantly less than WT mice at 18 and 24 weeks of age (*p*<.001 for both ages). The first death in R6/1 mice occurred at 18 weeks, and a total of 5 dead individuals were counted during the study (Fig 5B, Table 1). Two of them died following severe seizure during behavioral testing, while the remaining for unknown cause. First observation of clasping of hind limbs appeared in R6/1 animals at 14 weeks of age. Thereafter, the number of animals displaying this phenotype increased linearly reaching 77% at 24 weeks (Fig 5C).

R6/1 mice displayed shorter latencies to fall from grid throughout ages studied (*F*_1,104_ = 18.066, *p*<.0001, Fig 5D), differences in latencies appearing and accentuating at older ages (genotype x age interaction: *F*_4,104_ = 5.901, *p* = .0003), yielding significant differences between the two genotypes at 18 weeks (*p*<.05) and 24 weeks of age (*p*<.01). Performances of R6/1 mice in rotarod test were globally lower than those of WT mice (*F*_1,81_ = 26.167, *p*<.0001, Fig 5E), with significant differences between the genotypes at 18 weeks (*p*<.01) and 24 weeks (*p*<.01).

### Olfactory discrimination and detection

**Fig 6:**
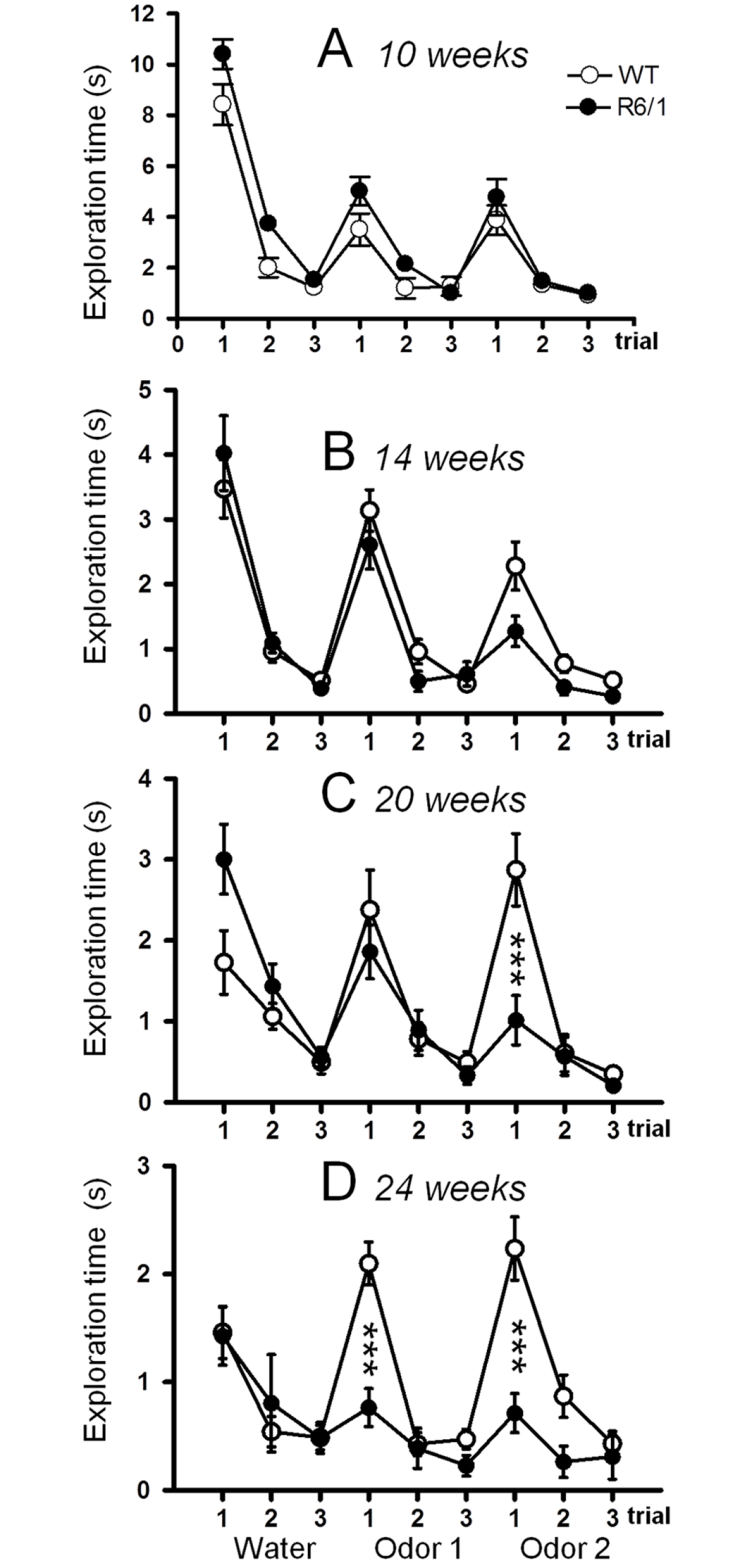
Olfactory detection and discrimination in R6/1 mice. *** R6/1 mice significantly different from age-matched WT littermates at *p*<.001.

No difference in the exploratory behavior towards the cotton applicator (with water) was observed between the two genotypes at 10 and 14 weeks (Fig 6A and 6B respectively): mice continued to equivalently display a strong exploratory behavior on the first trial whether water or an odor was presented, and then habituated with repeated presentation of the same odor-stimulus. The dishabituation indicated that animals of the two genotypes were able to distinguish between the different odors. It is noteworthy that R6/1 mice had a tendency to explore even more than WT mice at 10 weeks (*F*_1,22_ = 6.023, *p*<.05), but began to display a slightly decreased dishabituation at the second odor presentation at 14 weeks compared to WT littermates (trial 1 of odor 2).

An impairment in odor discrimination appeared in R6/1 mice at 20 weeks (odor x trial x genotype interaction: *F*_4,80_ = 5.092, *p*<.001, Fig 6C). R6/1 mice were not able to distinguish between the two different odors, as they did not display dishabituation toward the second odor presentation as did WT mice (genotype: *t*_20_ = 3.656, *p* = .002). The deficits in R6/1 mice became more severe at 24 weeks (genotype x odor interaction: *F*_2,64_ = 7.055, *p*<.01; genotype x trial interaction: *F*_2,64_ = 8.076, *p*<.001, Fig 6D), since not only they weren’t able to distinguish between odor 1 and odor 2, as indicated by the absence of dishabituation at the first trial of odor 2 (genotype: *t*_16_ = 4.443, *p*<.001), but also they did not present any dishabituation at the first presentation of odor 1 (genotype: *t*_16_ = 4.881, *p*<.001).

### Social behaviors and vocalisations

**Fig 7:**
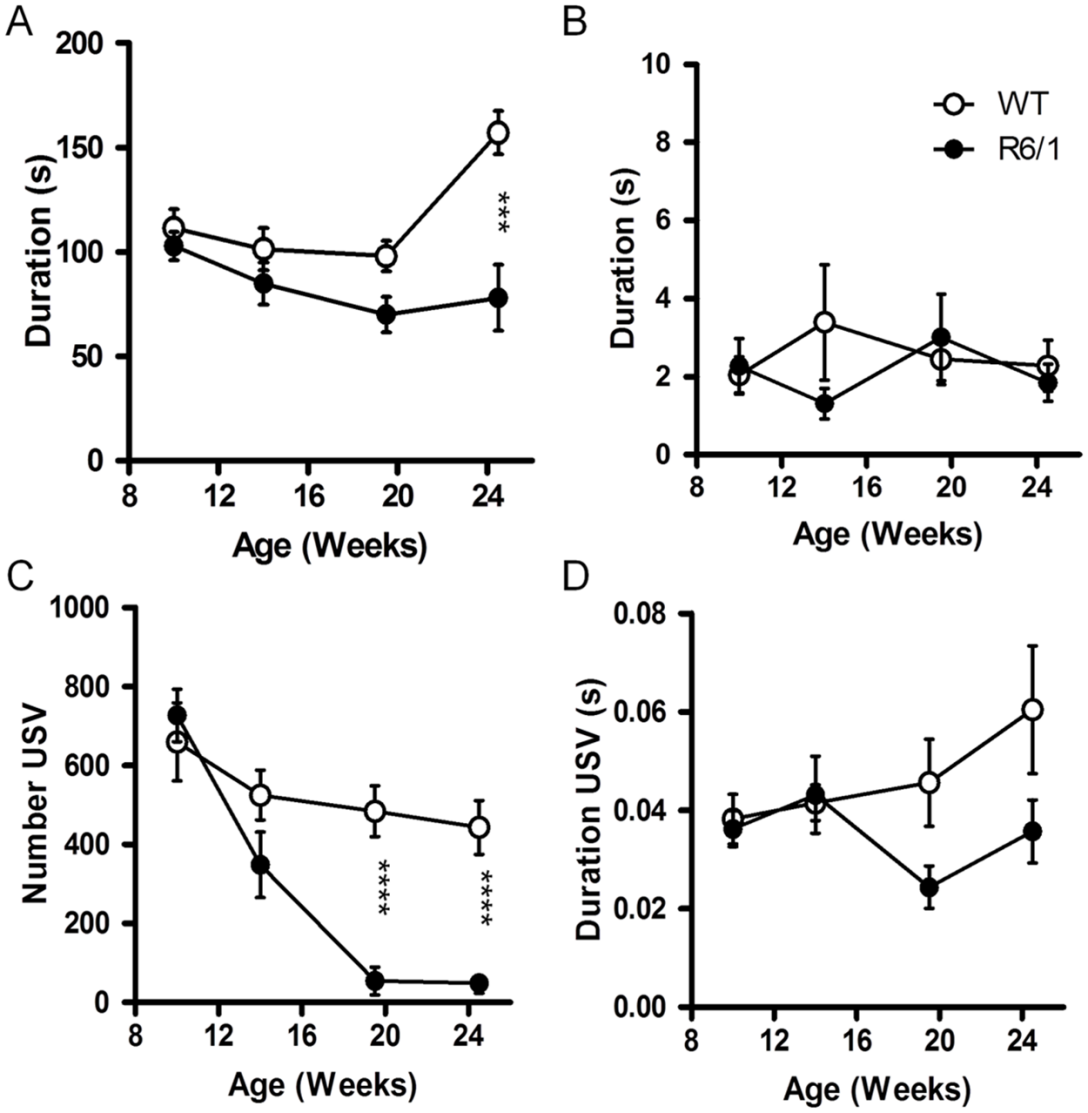
Social behaviors in R6/1 mice. Time spent in social affiliative behaviors (A) and non social behaviors (B), number of ultrasonic vocalization (C) and their mean duration (D) in both groups of mice at different ages. *** R6/1 mice significantly different from age-matched WT littermates at *p*<.001.

R6/1 mice displayed a reduction in social behaviors across ages (*F*_1,81_ = 20.81, *p*<.0001, Fig 7A), while such difference was not found for non-social behaviors (*F*_1,81_<1, n.s. Fig 7B). The time spent in social behaviors was different between genotypes at 24 weeks (p<.001), but not at younger ages (p>.05, genotype x age interaction: *F*_3.81_ = 4.51, *p*<.01).

In addition, during social interaction, R6/1 mice emitted less ultrasonic vocalisations than WT in general (Fig 7C, *F*_1,67_ = 10.95, *p*<.01). The number of vocalisation decreased with age (*F*_3,67_ = 14.17, *p*<.0001), and this in particular in R6/1 mice (genotype x age interaction: *F*_3,67_ = 5.29, *p*<.01). Significant differences between the genotypes were found at 20 and 24 weeks of age (p<.0001 for both comparisons). However, the mean duration of vocalisation was only slightly decreased in R6/1 mice (*F*_1,67_ = 4.34, *p*<.05, Fig 7D), in an age-independent manner (genotype x age interaction: *F*_3,67_ = 4.44, n.s.).

### Reinforced spatial alternation

**Fig 8:**
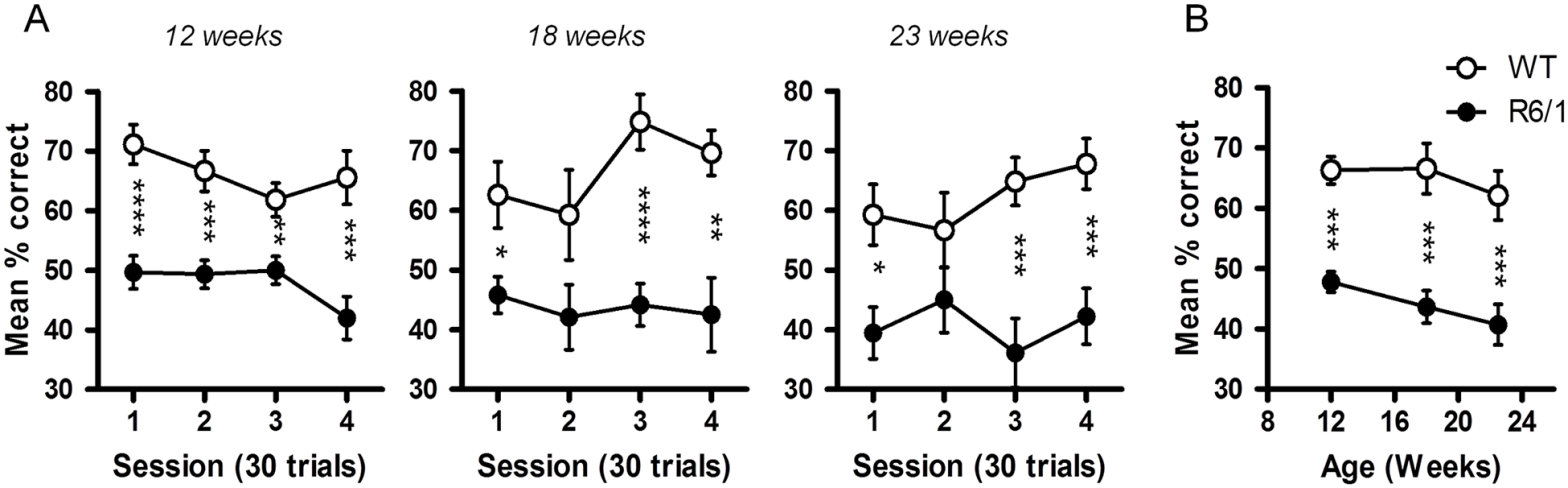
Reinforced spatial alternation in T-maze in R6/1 mice. Mean (SEM) percent correct for 4 consecutive sessions (30 trials/session) at 12, 18 and 23 weeks of age (A) and averaged scores for 4 sessions at each age (B). * R6/1 mice significantly different from age-matched WT littermates at *p*<.05, ** at *p*<.01, *** at *p*<.001 and **** at *p*<.0001.

R6/1 mice alternated significantly less than age-matched WT littermates at each age studied (*F*_1,17_ = 44.00, *p*<.0001 at 12 weeks, *F*_1,15_ = 20.09, *p*<.001 at 18 weeks, *F*_1,13_ = 14.06, *p*<.01 at 23 weeks, Fig 8A). The reduction of alteration rates was identical throughout 4 sessions performed at all ages. Similarly, R6/1 mice displayed significantly lower levels of averaged alternation across different ages (*F*_1,13_ = 29.86, *p*<.0001), the magnitude of the impairment remaining identical at all ages (genotype x age interaction, *F*_2,26_<1, n.s., Fig 8B).

## Discussion

Here we report an early deterioration of sleep physiology which is timely correlated with spatial working memory impairments in R6/1 mice. These sleep and cognitive impairments were followed by sensory, motor and social behavioral disturbances, revealing that sleep physiology changes are one of early phenotypic alterations in our HD mouse model (Table 2).

**Table 2:** Summary of multi-domain phenotypic changes across age in R6/1 mice.

Age (weeks)	Phenotypes in R6/1 mice
9–12	Delta power decreaseSleep fragmentationSpatial working memory impairment
13–16	Breakdown of circadian rhythm
17–20	Motor coordination and grip strength deficits, ClaspingDeficits in olfactory discriminationBody weight loss
21–24	Deficits in odor detectionDeficits in social behavior and ultrasonic vocalization

### Sleep changes in HD mice

Although the striatum and cortex are particularly vulnerable in early HD, there also are significant pathological alterations of the hypothalamus and brainstem, brain regions that are critically involved in fundamental behaviors including sleep-wake and circadian rhythm regulation [23,24,25,26]. Coherently, data presented here highlight and designate the sleep dysregulation as early phenotypic changes appearing at motor pre-symptomatic ages in R6/1 mice. More precisely, R6/1 mice at 11 weeks displayed fragmented sleep with increased sleep-wake transitions and decreased sleep and waking durations. Curiously, the sleep fragmentation did not result in major changes in total sleep and waking durations in proportion, preserving somewhat normal sleep structures at this young age. A further dysregulation at 16 weeks concerned a decreased waking and an increased sleep time during dark cycle, abolishing activity difference between light and dark cycles in R6/1 mice. The sleep fragmentation at 11 weeks coincides in time with severe cognitive impairments assessed in a spatial working memory test. R6/1 mice were incapable of alternating between left and right arms in a T-maze based on their short-term working memory. Furthermore, the circadian rhythm breakdown at 16 weeks “announces” abnormalities in other sensory, motor and psychiatric domains. In line with this idea, therapeutic attempts by managing sleep-wake cycles using either hypnotics or scheduled feeding improved not only sleeping, but also other behavioral and cognitive abnormalities in R6/2 mice [27]. This observation suggests the idea that sleep-wake changes may contribute significantly to several other HD symptoms.

### Comparison with HD patients

While longitudinal sleep studies from pre- to post-symptomatic stages in HD patients have not been reported, some discrepancies exist when comparing data from humans and transgenic mice. For example, patients at early phases spend more time in SWS [28] and less time in REM sleep [29,30,31], while our R6/1 and R6/2 mice at motor asymptomatic stage spent less time in SWS [13] and more time in REMs [13,14]. HD patients displayed increased wakefulness at resting periods [5,28,29,31,32], but not in our mice. However, both patients and R6/1 (and R6/2) mice displayed (1) sleep fragmentation [5,6,7,12,32], (2) reduced total sleep time [29] and (3) daytime somnolence in patients [33,34] and frequent sleep at dark cycle in mice. This indicates that although not all characteristics of sleep perturbation were reproduced, there exists a great deal of similarity between human and mouse sleep disturbances in HD.

### Sleep spectral changes in delta frequency in R6/1 mice

Additional spectral analyses of sleep recordings revealed severe and early changes of delta (1–4 Hz) synchrony (12 weeks) in R6/1 mice, age at which severe cognitive impairments were observed in these mice. The magnitude of the delta power reduction increased with age of the transgenic mice. These results confirm a recent observation made in R6/2 mice with 250 CAG repeats [14].

This slow oscillation setting so called “up and down states” is known to arise within the thalamo-cortical circuit, and the thalamus also undergoes degeneration in HD [35,36,37]. Therefore, the thalamo-cortical abnormalities might be an important event for the HD pathophysiological progression. The up and down states are known to facilitate information transfer among subcortical-cortical regions by setting time favourable for synchrony, thereby modulating memory consolidation process [38,39,40]. The impoverishment of delta power during SWS seen in our transgenic mice may thus contribute to their cognitive impairments. In support of this notion, our previous study [17] revealed that R6/1 mice were slower in acquiring procedural operant conditioning. More precisely, the altered behaviors may be related to inability of these mice of transferring learned information across days because mice re-acquired freshly in each session.

Our spectral data confirm and compliment three recent works including ours, reporting major changes in low gamma/beta frequencies (25–60 Hz) during SWS and REM sleep in both R6/1 and R6/2 mice [13,14,15] as well as unusual theta expression during SWS in symptomatic R6/1 mice [41].

### Social behavioral and olfactory deficits in R6/1 mice

Sleep disturbed R6/1 mice were also impaired for olfactory functions, although the abnormalities appeared later and their severity increased with age. More precisely, progressive olfactory deterioration occurred in selective discrimination between two odors at 20 weeks of age which worsened to odor detection deficits at 24 weeks of age. These olfactory dysfunctions were not due to a limited tendency to explore environmental stimuli of transgenic mice, because their exploration (even when no odor was presented) remained intact throughout aging, or even enhanced at some age points. The olfactory deficits have been previously shown in R6/1 and R6/2 mice as well as in HD patients associated with the deterioration of structural plasticity marker and neurogenesis in the primary olfactory cortex [42,43,44,45,46,47].

Interestingly, at 20 weeks social behavioral changes also emerged; this is later than what detected in our previous study [18]. It is possible that the individual housing condition in the present experiment, rather than the grouped housing used previously, may have increased the social interest of the animals in general, and have masked the potential deficits in the transgenic mice at earlier ages. A second possibility relies on the repeated exposure to behavioral testing that may represent a form of environmental enrichment, known to have protective effect against neuropathology in general, including HD [48,49,50]. The same comment could also be formulated to all the tests administered repeatedly over the 4 months period. Social behavioral changes are reminiscent of apathy and social withdrawal observed in HD patients at early phase of the pathology [51,52], and may be associated with striatal dysfunction [53]. However, olfactory deficits observed simultaneously might have interfered with social behaviors, which greatly rely on olfactory exploration (sniffing). Nonetheless, we believe this is unlikely at least for the early ages, i.e., 20 weeks, when R6/1 mice actually explored even more than WT a novel olfactory stimulus.

## Conclusion

Here, we report the outcome of the first study designed to monitor changes in the quality and quantity of sleep in parallel with the determination of the onset and progression of multi-domain phenotypes in R6/1 mice. Our data suggest severe brain network activity changes during sleep-wake cycles at early age (i.e. 11 weeks). In addition, sleep spectral change will be a useful in vivo biomarker with which the efficacy of innovative therapeutics against HD could be tested.
